# Influence of NH_3_ plasma and Ti doping on pH-sensitive CeO_2_ electrolyte-insulator-semiconductor biosensors

**DOI:** 10.1038/s41598-017-02692-2

**Published:** 2017-05-25

**Authors:** Chyuan-Haur Kao, Che-Wei Chang, Yu Tzu Chen, Wei Ming Su, Chien Cheng Lu, Chan-Yu Lin, Hsiang Chen

**Affiliations:** 1 Department of Electronic Engineering, Chang Gung University, Taoyuan, 333 Taiwan, ROC; 2 Department of Applied Materials and Optoelectronic Engineering, National Chi Nan University, Puli, 545 Taiwan, ROC; 3 Kidney Research Center, Department of Nephrology, Chang Gung Memorial Hospital, Chang Gung University, College of Medicine, Taoyuan, Taiwan, ROC; 4 Department of Electronic Engineering, Ming Chi University of Technology, New Taipei City, Taiwan, ROC

## Abstract

In this study, CeO_2_ pH-sensitive sensing membranes in electrolyte-insulator-semiconductor structures on silicon substrate were fabricated. To enhance sensing performance, the membrane underwent Ti doping and NH_3_ plasma treatment on the surface. To examine the effects of Ti doping and plasma treatment, multiple material properties evaluations were conducted using field-emission scanning electron microscopy, X-ray diffraction, atomic force microscopy, and secondary ion mass spectroscopy. Results indicate that Ti doping and plasma treatment can remove defects and enhance crystallization, thereby achieving improved pH-sensing performance of the membrane with high sensitivity, high linearity, low hysteresis voltage and low drift voltage. CeO_2_-based EIS membranes with Ti doping and NH_3_ plasma treatment show promise for future portable pH-sensitive biosensors.

## Introduction

Within this decade, pH-sensing technologies have been intensively studied for biochemical applications. This is due in large part to the fact that pH value is key to the health of living organisms, influencing function, development and growth of living systems. In the early 20th century, researchers have used colorimetric and electrometric methods to examine the pH values in different solutions by observing their colors and measuring their voltage variations^1^. Since then, light detection and voltage detection have become two distinct methods by which to carry out pH sensing^2^. For pH sensing using the light detection approach, optical fibers were utilized to monitor color changes of the dye in solutions in the 1980s^3^. Recently, Li *et al*. used detection of fluorescent light absorption related to energy level variations to evaluate pH values^4^. Alternately, detection of voltage variation from a semiconductor device began from 1970, when Bergveld invented the first ion-sensitive field effect transistor (ISFET)^5^. An ISFET is derived from a metal oxide semiconductor field effect transistor with an ion-sensing gate structure, in contact with a buffer solution. In the 1990s an electrolyte-insulator-semiconductor (EIS) structure was invented using a sandwiched insulating sensing membrane in contact with an electrolyte on top and a semiconductor on the bottom^6^. EIS biosensing devices have been attracting intensive attention because of their rapid response, robustness, compact size, and possible integration with an on-chip circuit^7, 8^. Over the past decade, various types of metal oxides such as Nb_2_O_5_^9^, HfO_2_^10^, and TiO_2_^11^ have been used as the sensing insulator in an EIS structure. Recently, some rare earth oxides, with advantages including wide band gaps, large band offsets on Si, and high dielectric constants have been demonstrated as good sensing insulators for EIS biosensing devices^12, 13^. Among these rare earth oxides, CeO_2_, with a wide bandgap of 3.19 eV and a high dielectric constant, has been used as the sensing material for EIS biosensing devices^14, 15^. In addition, Kao *et al*. have proposed the positive effects of annealing on the CeO_2_ membrane in 2014^16^, and the influence of CF_4_ plasma treatment on the CeO_2_ sensing insulator in 2015^17^. However, to further improve the material properties of the sensing membrane and hence boost the sensing capability, alternative processes or distinctive treatments are worth exploration and investigation. In addition to the conventional thermal annealing treatment, incorporation of atoms during membrane layering by co-sputtering^18^ or the addition of different atoms after membrane deposition with plasma treatment^19^ have been proposed to reinforce the membrane in order to reducing defects. Recently, the incorporation of Ti atoms^20^ and NH_3_ plasma treatment^21^ have been utilized to improve the sensing membrane performance. Based on the previous report^20^, Ti doping in the insulator layer can fix defects in the membrane^22^. Furthermore, addition of N atoms through the NH_3_ plasma treatment on the surface of the sensing insulator can mitigate dangling bonds on the membrane surface and hence ameliorate the solution/insulator interface during the sensing operation^23, 24^. In this paper, we combined Ti doping and NH_3_ plasma treatment to optimize the sensing performance of the membrane.

In this research, CeO_2_ membrane-based EIS biosensors were fabricated with Ti doping into the membrane by cosputtering and N-atom incorporation by NH_3_ plasma treatment on the surface of the membrane. Moreover, multiple material analyses, including secondary ion mass spectroscopy (SIMS), field-emission scanning electron microscopy (FESEM), X-ray diffraction (XRD), and atomic force microscopy (AFM) were performed to study the improvements of material properties caused by Ti doping and NH_3_ plasma treatment. SIMS results show that the piling up of N atoms may fix interfacial dangling bonds. Moreover, FESEM images clearly indicate that both Ti doping and NH_3_ plasma treatment can enhance granization. Consistent with the FESEM images and XRD analysis, AFM images reveal that incorporation of Ti atoms can reinforce the crystallization of the CeO_2_ insulator. In addition, the pH-sensing sensing capabilities were measured^25, 26^. In line with the material analysis, results indicate that incorporation of Ti doping and NH_3_ plasma treatments could effectively improve pH sensing behavior and sensing capability. The Ti-doped NH_3_ plasma treated EIS biosensors have the potential to develop future portable biochemical sensors at an industrial level.

## Results and Discussion

**Figure 1:**
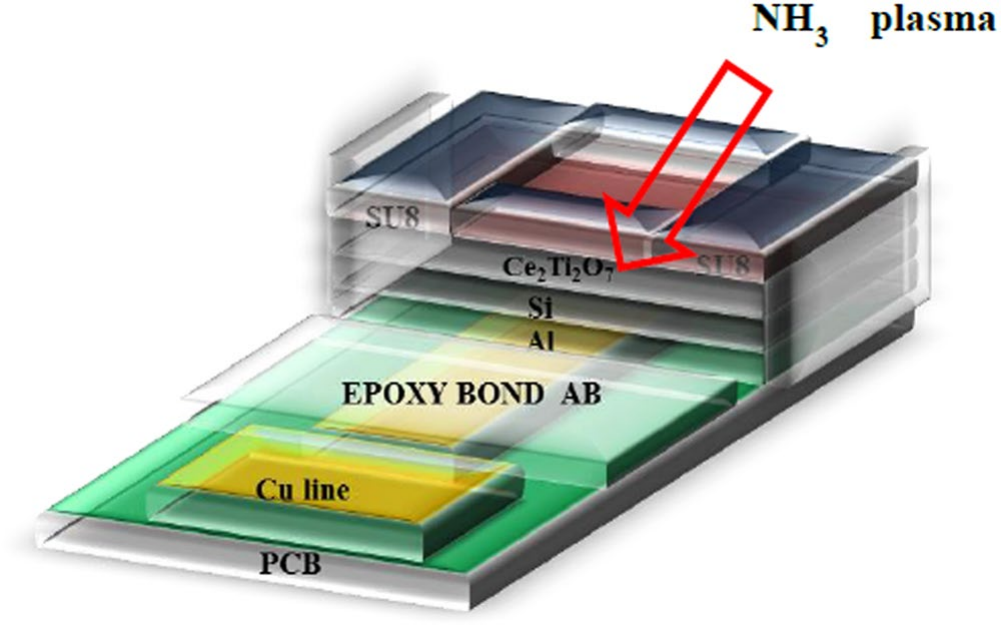
The Ce_2_Ti_2_O_7_ EIS structure.

**Figure 2:**
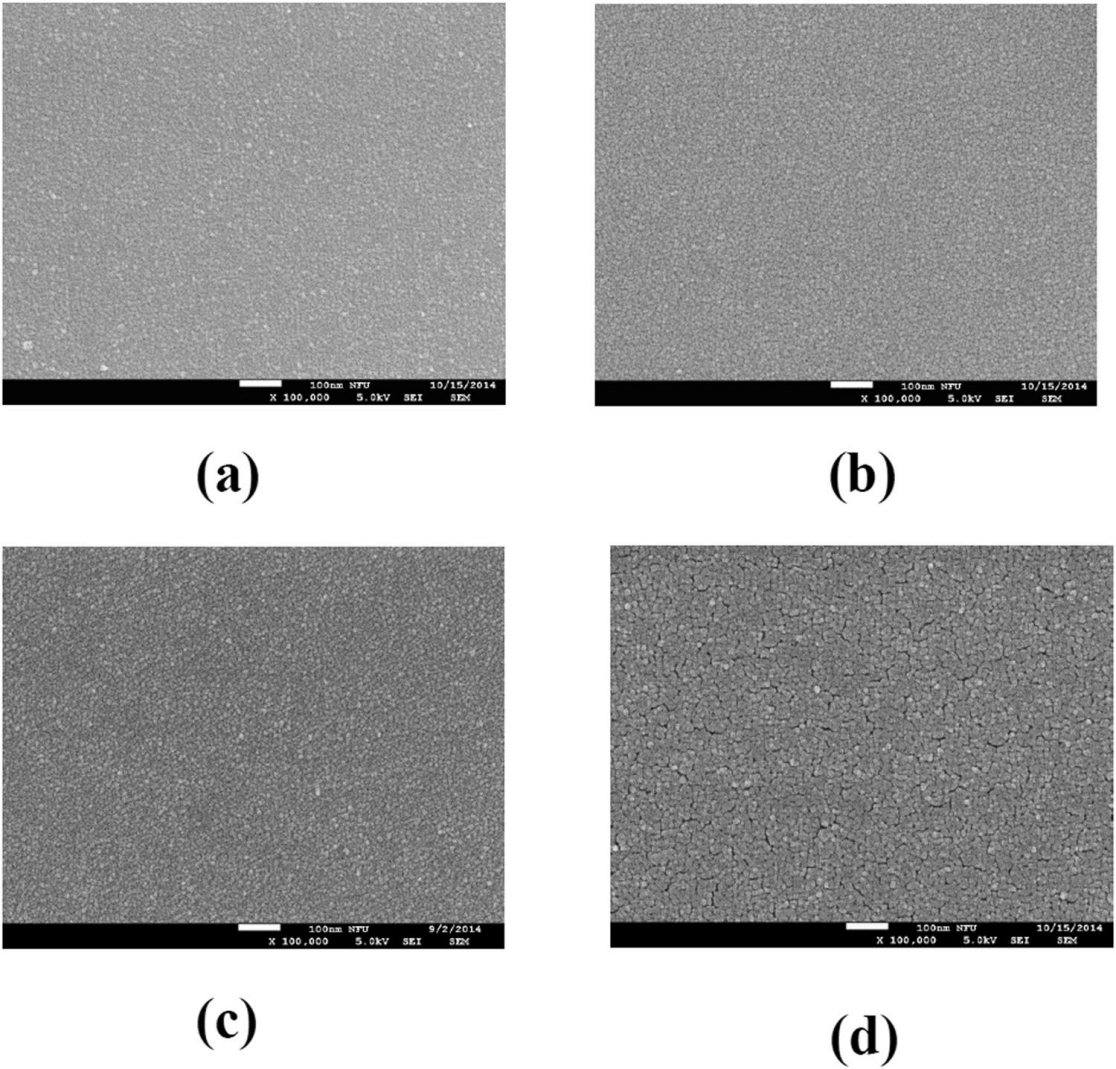
FESEM images of (**a**) the as-deposited CeO_2_ sample (**b**) the Ce_2_Ti_2_O_7_ sample (**c**) the as-deposited CeO_2_ sample with NH3 plasma treatment for 3 min (**d**) the Ce_2_Ti_2_O_7_ sample with NH_3_ plasma treatment for 3 min.

The detailed EIS structure is illustrated in Fig. 1. Material analyses were performed on the membrane film and sensing measurements were conducted on the EIS sensors. To characterize the influence of Ti addition and NH_3_ plasma treatment on the CeO_2_ membrane, multiple material characterizations including FESEM, XRD, AFM, and SIMS were performed on CeO_2_ and Ce_2_Ti_2_O_7_ films with and without NH_3_ plasma treatment. First, we used FESEM to view the surface morphologies of CeO_2_ and Ce_2_Ti_2_O_7_ films as shown in Fig. 2(a,b,c and d). An FESEM image of the as-deposited CeO_2_ film is shown in Fig. 2(a). Compared with the as-deposited CeO_2_ film, the FESEM image of the Ti-doped CeO_2_ film without NH_3_ plasma treatment as shown in Fig. 2(b) reveals that clearer nanograins could be observed, indicating that Ti addition might reinforce crystallization. Similarly, the CeO_2_ film treated with NH_3_ plasma as shown in Fig. 2(c) exhibited clearer grain images than the CeO_2_ as-deposited film, showing that NH_3_ plasma treatment could enhance crystallization as well. Furthermore, CeO_2_ film incorporating Ti atoms with NH_3_ plasma treatment as shown in Fig. 2(d) exhibited the strongest crystallization among all the samples. In addition, slight cracks around the grains might further enhance the contact area in the membrane/electrolyte surface and boost the sensing performance.

**Figure 3:**
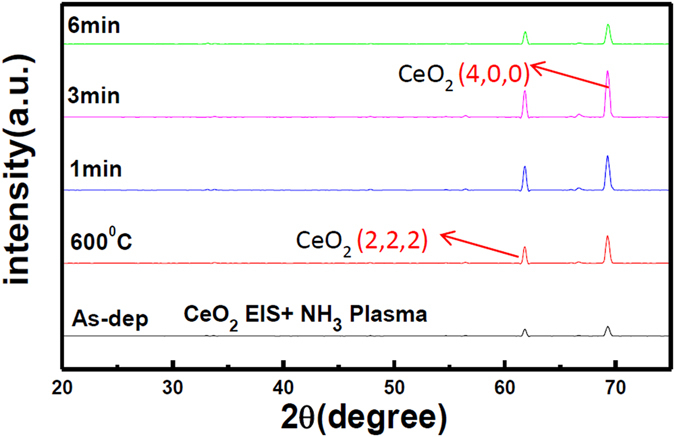
XRD of (**a**) CeO_2_ samples with NH3 plasma treatment in various conditions. (**b**) Ce_2_Ti_2_O_7_ samples with NH3 plasma treatment for 3 min.

Additionally, we used XRD to examine the CeO_2_ films and the Ce_2_Ti_2_O_7_ films incorporated with Ti atoms in various NH_3_ plasma treatment conditions, as shown in Fig. 3(a and b). Consistent with the FESEM images as shown in Fig. 2(a,b,c and d), XRD patterns reveal that NH_3_ plasma treatment for 3 min could drastically enhance the CeO_2_ (400) peak and the CeO_2_ (200) peak intensity indicative of crystallization during NH_3_ plasma treatment. Furthermore, Ti addition could cause CeO_2_ crystals to form Ce_2_Ti_2_O_7_ crystals as shown in Fig. 3(b). Furthermore, NH_3_ plasma treatment for 3 min could enhance crystallization as 3 min NH_3_ plasma treatment increased the Ce_2_Ti_2_O_7_ (2,2,1) peak and the Ce_2_Ti_2_O_7_ (2,1,2) peak drastically. The results show that Ti addition and NH_3_ plasma treatment could effectively strengthen crystallization and hence improve the sensing performance of the CeO_2_ membrane.

**Figure 4:**
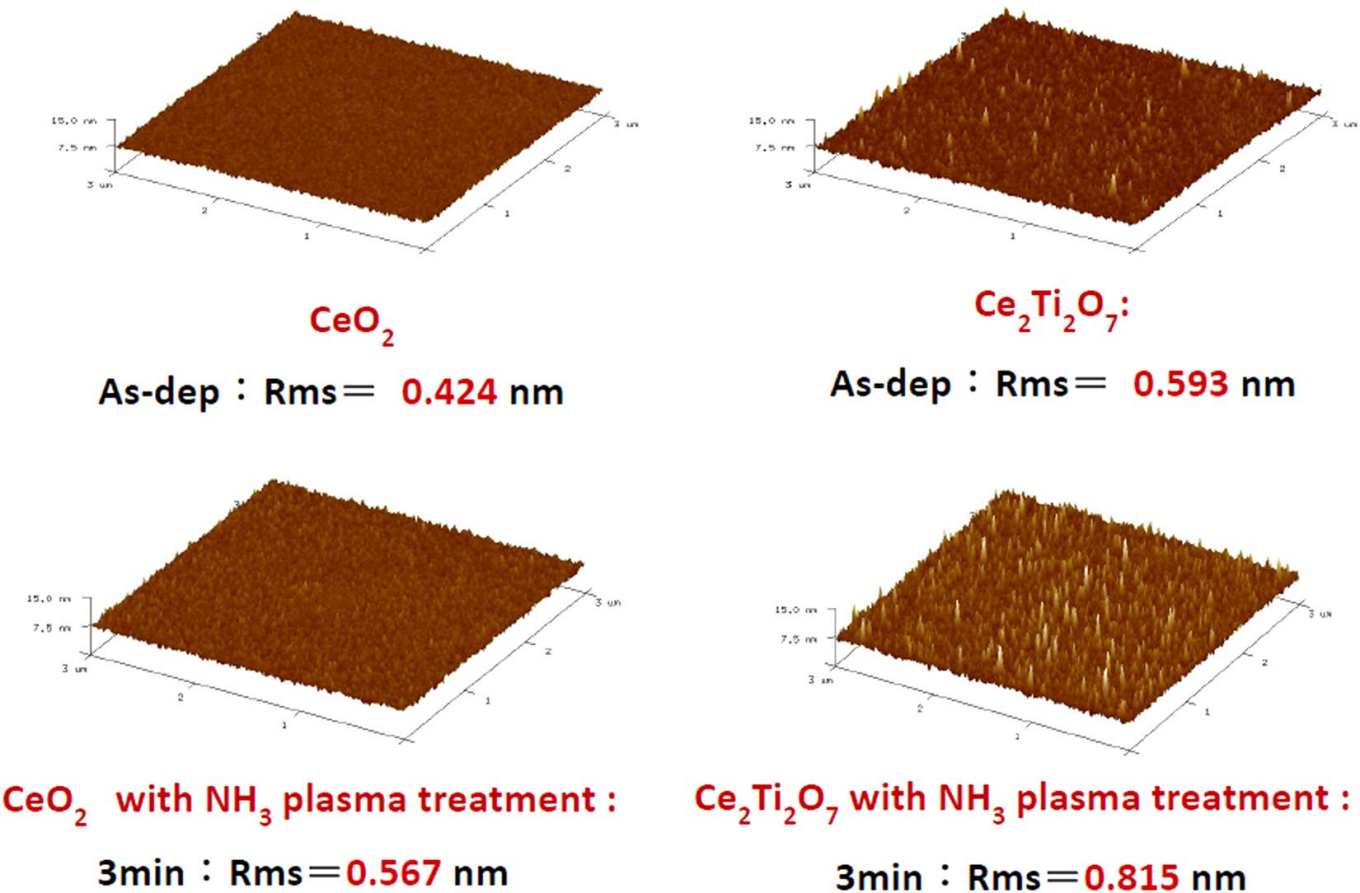
AFM images of the CeO_2_ sample without NH_3_ plasma treatment and with NH_3_ plasma treatment for 3 min. The normalized C-V curve of the Ce_2_Ti_2_O_7_ sample without and with NH_3_ plasma treatment for 3 min.

In addition, AFM images as shown in Fig. 4 reveal that the surface roughness of the CeO_2_ and Ce_2_Ti_2_O_7_ films treated in various NH_3_ plasma conditions. Compared with the CeO_2_ film and Ce_2_Ti_2_O_7_ as shown in Fig. 4, Ti doping could effectively increase the surface roughness and cause the grain on the surface to become more noticeable. Similarly, NH_3_ plasma treatment could increase roughness and cause grain growth as well. Moreover, incorporating both Ti doping and NH_3_ plasma treatment for 3 min could drastically increase the roughness from 0.424 nm to 0.815 nm and clearly enhance the grains, as shown in the AFM images.

**Figure 5:**
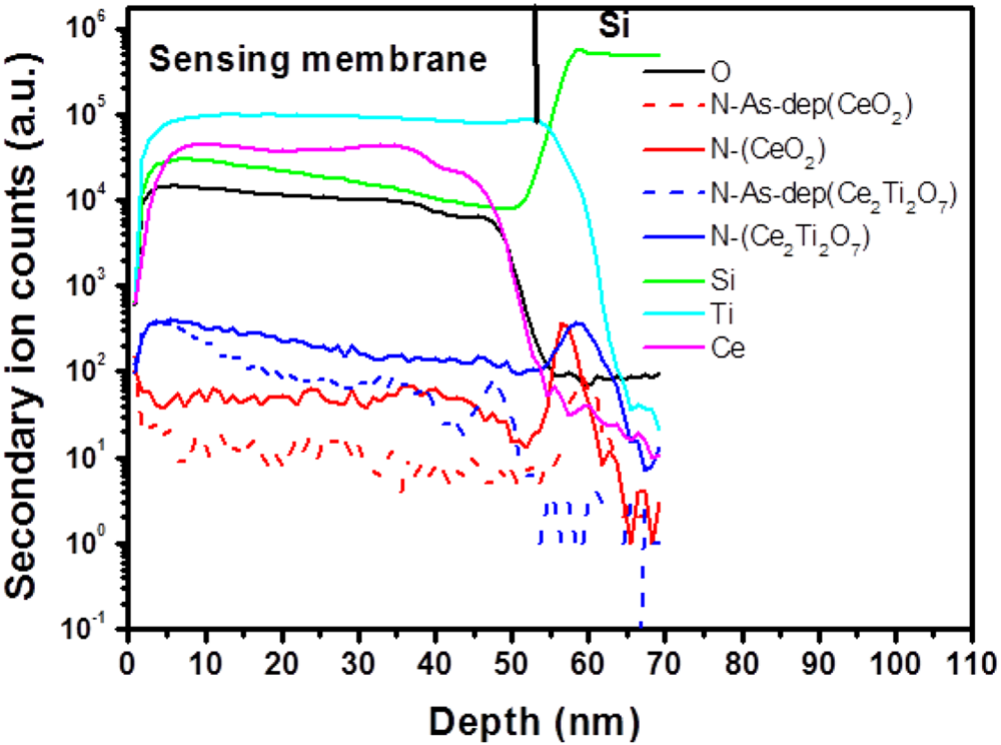
SIMS profiles of CeO_2_ or Ce_2_Ti_2_O_7_ sensing membrane/Si with NH_3_ plasma treatment.

Furthermore, SIMS analysis as shown in Fig. 5 reveals the distribution of various atoms inside the CeO_2_/Si film and the Ce_2_Ti_2_O_7_/Si films. Figure 5 shows stronger N atom concentrations in both the CeO_2_ film and the Ce_2_Ti_2_O_7_ film after NH_3_ plasma treatment. Moreover, noticeable accumulation could be observed in the membrane/Si interface. Since NH_3_ plasma treatment could infuse N atoms into the CeO_2_ and Ce_2_Ti_2_O_7_ films, stronger N-Ce and N-Si bonds might be formed and dangling bonds or defects around the interface might be fixed^19, 23^. Strengthening the material quality in the bulk and in the interface might increase the sensitivity and linearity of pH sensing of the films. Furthermore, a high concentration of Ti atoms in the SIMS profile for the Ce_2_Ti_2_O_7_ films could be observed. Since Ti addition could also fix traps and dangling bonds, a high concentration of Ti atoms in the Ti-doped CeO_2_ film could cause the Ti atoms to reduce the defects^18^. In addition, a slight increase of concentration of Ti atoms might indicate the accumulation of the Ti atoms around the interface and fix the dangling bonds near the interface.

Incorporating a CeO_2_ membrane as the electroactive gate film in the pH-based EIS structure allows the incorporated sensor to detect fine pH value variations. The influence of the plasma treatment and Ti doping can be realized by studying the site binding model^27^. The reference voltage is closely related to the surface potential, which is dependent on the pH value of the electrolyte and the membrane material.

1 $$\psi =2.303\frac{kT}{q}\frac{\beta }{\beta +1}(p{H}_{pzc}-pH)$$

The ${\rm{\phi }}$ value can be calculated from the above equation (1), where k is the Boltzmann’s constant, T is the temperature, and ${\rm{\beta }}$ is a parameter in terms of the chemical sensitivity of the membrane^28–30^. The value of ${\rm{\beta }}$ is proportional to the density of surface hydroxyl groups and is determined by the following equation (2).

2 $${\rm{\beta }}=\frac{2{q}^{2}Ns\sqrt{{K}_{a}{K}_{b}}}{KT{C}_{DL}}$$

N_s_ is the number of surface sites per unit area. K_a_ is the equilibrium constant of the acid point and K_b_ is the equilibrium constant of the base point, respectively. C_DL_ is the bilayer capacitance calculated from the Gouy-Chapman-Stern model^31^.

**Figure 6:**
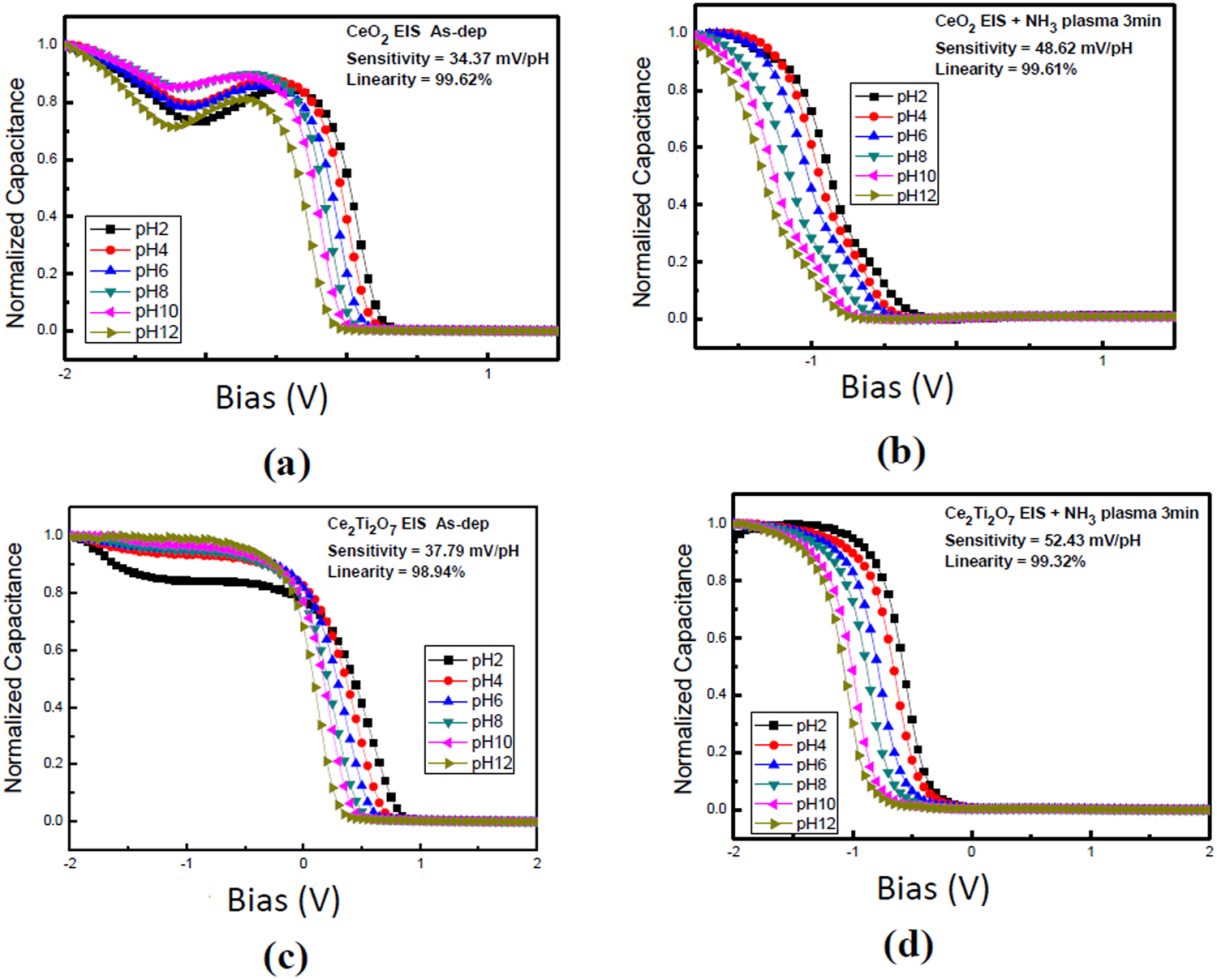
The normalized C-V curve of the CeO_2_ sample (**a**) without NH_3_ plasma treatment and (**b**) with NH_3_ plasma treatment for 3 min. The normalized C-V curve of the Ce_2_Ti_2_O_7_ sample (**c**) without and (**d**) with NH_3_ plasma treatment for 3 min.

**Figure 7:**
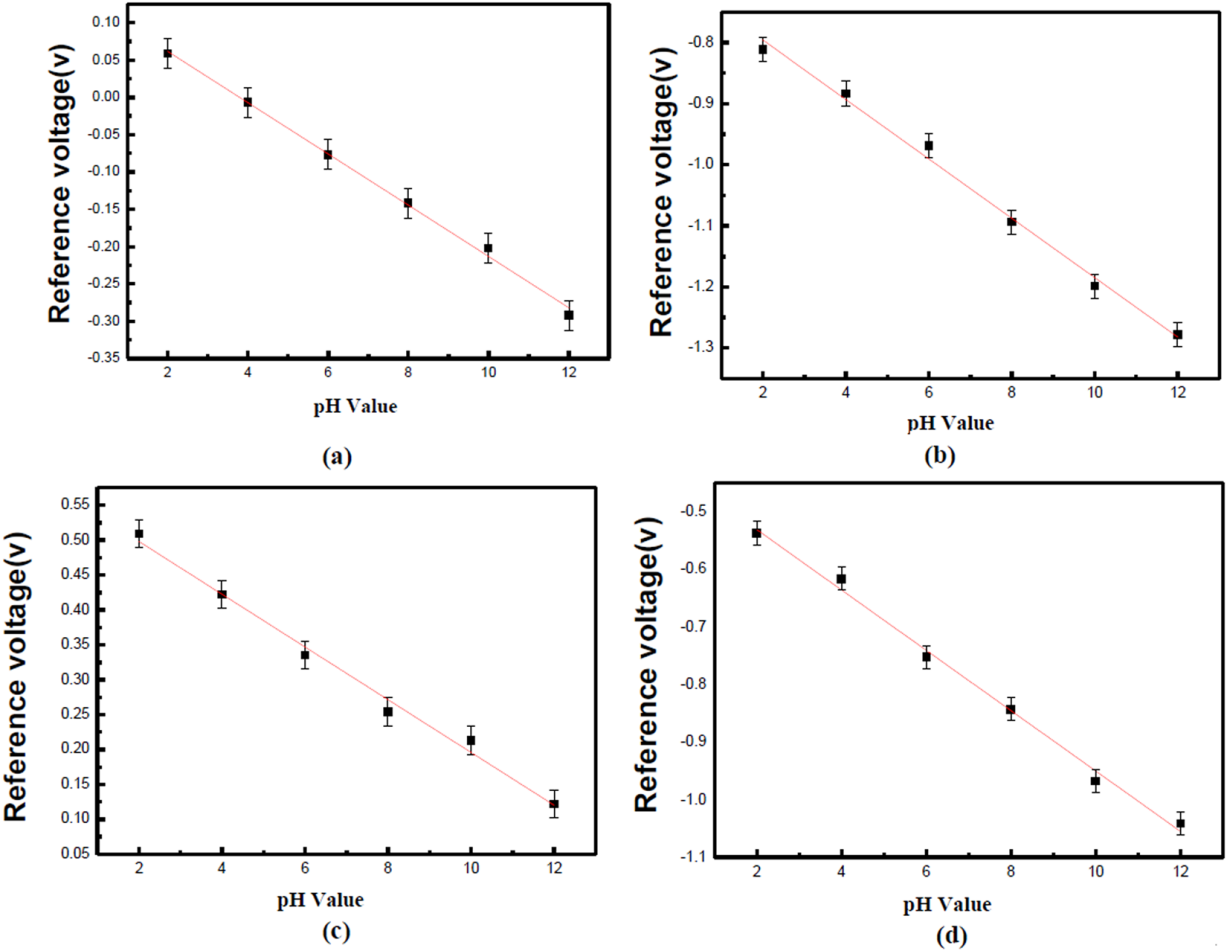
The linearity and sensitivity extracted from normalized C-V curve for the CeO_2_ sample (**a**) without NH_3_ plasma treatment and (**b**) with NH_3_ plasma treatment for 3 min. The normalized C-V curve of the Ce_2_Ti_2_O_7_ sample (**c**) without NH_3_ plasma treatment and (**d**) with NH_3_ plasma treatment for 3 min.

According to the above theories, addition of Ti atoms and incorporation of NH_3_ plasma treatment could enhance crystallization and reduce the dangling bonds. Therefore, the number of the surface sites could be increased and the sensing performance could be boosted. To characterize the sensing performance of the CeO_2_ and Ce_2_TiO_7_ membranes with and without NH_3_ plasma treatment, the C-V curves of these various membranes are shown in Fig. 6(a,b,c and d). The linearity and sensitivity of the CeO_2_ and Ce_2_TiO_7_ membranes with and without NH_3_ plasma treatment extracted from normalized C-V curve are shown in Fig. 7(a,b,c and d). As shown in Fig. 6(a) and 7(a), the as-deposited CeO_2_ membrane exhibited a low sensitivity of 34.37 mV/pH. Moreover, fluctuated C-V curves could be observed indicating multi-capacitance effects were present, signifying that defects in the bulk or in the interface might be present. As the membrane underwent the NH_3_ plasma treatment for 3 min, the sensitivity was boosted to 48.62 mV/pH and the C-V curves became smoother as shown in Figs 6(b) and 7(b). Similarly, as the as-deposited CeO_2_ membrane and the as-deposited Ce_2_TiO_7_ membrane were compared as shown in Fig. 7(a and c), the sensitivity was improved when the Ti atoms were incorporated into the CeO_2_ membrane. Furthermore, as the Ce_2_TiO_7_ membrane went through NH_3_ plasma treatment for 3 min, the sensitivity was greatly improved to 54.43 mV/pH. Furthermore, much smoother C-V curves could be observed, indicating single capacitance with high material quality film could be formed. Combining Ti doping and NH_3_ plasma treatment could enhance the CeO_2_ film material quality and sensing capability.

**Figure 8:**
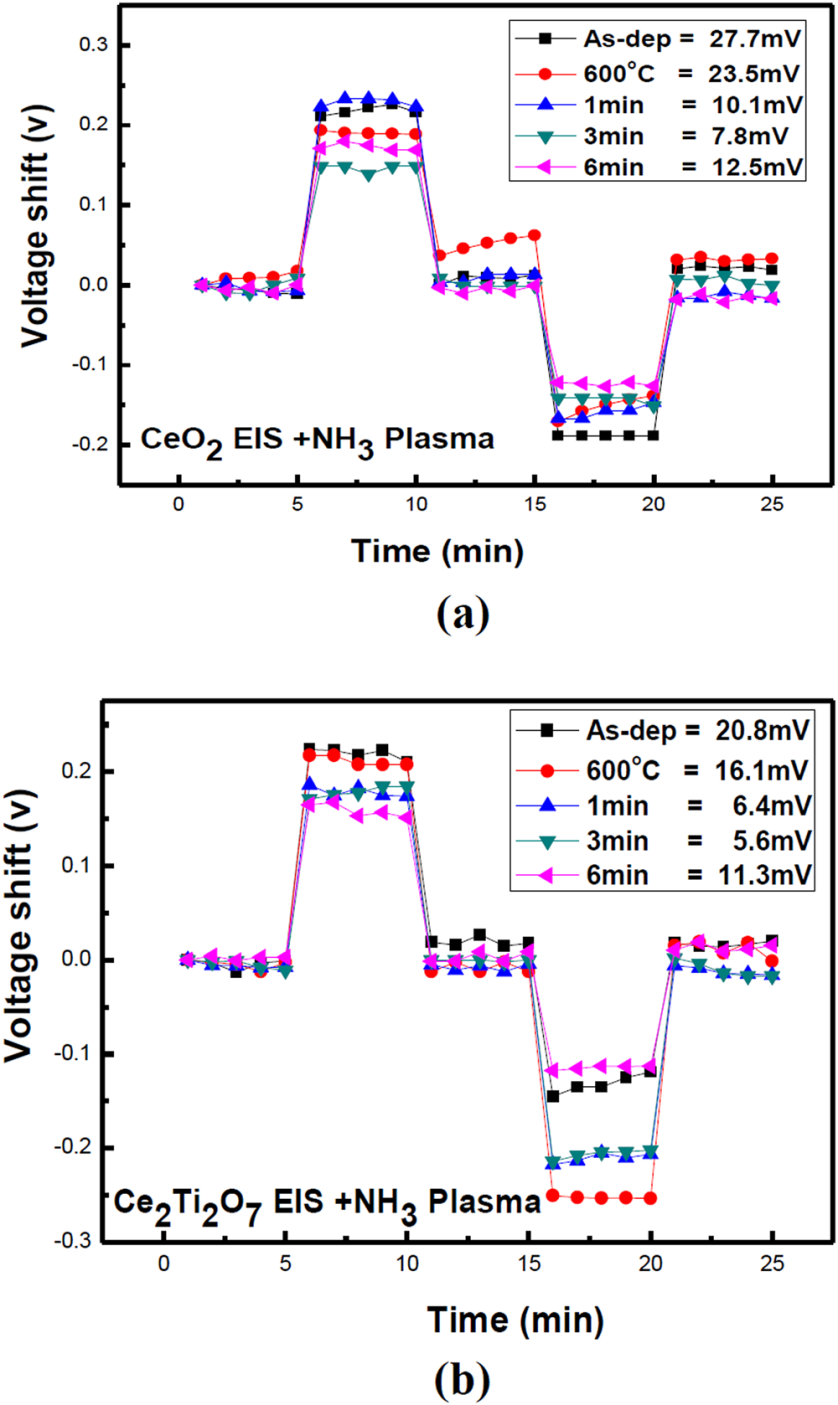
The hysteresis of (**a**) CeO_2_ and (**b**) Ce_2_Ti_2_O_7_ sensing membrane with NH_3_ plasma treatment in various conditions during a pH loop of 7→4→7→10→7 over a period of 25 minutes.

Furthermore, to study the hysteresis effects of the CeO_2_ and Ce_2_TiO_7_ films which underwent through various plasma treatment conditions, hysteresis voltages were measured for the CeO_2_ film and the Ce_2_Ti_2_O_7_ film with different plasma treatment conditions, as shown in Fig. 8(a and b). The as-deposited CeO_2_ film with a hysteresis voltage of 27.7 mV and the as-deposited Ce_2_Ti_2_O_7_ film with a hysteresis voltage of 20.8 mV could be observed, indicating that Ti-doping might reduce the dangling bonds and traps to lower the hysteresis voltage. Furthermore, the CeO_2_ with NH_3_ plasma treatment had a low hysteresis voltage of 7.8 mV, and the Ce_2_Ti_2_O_7_ film with NH_3_ plasma voltage had the lowest hysteresis voltage of 5.6 mV. The results indicate that NH_3_ plasma treatment could passivate defects and enhance sensing performance, consistent with the material analysis.

**Figure 9:**
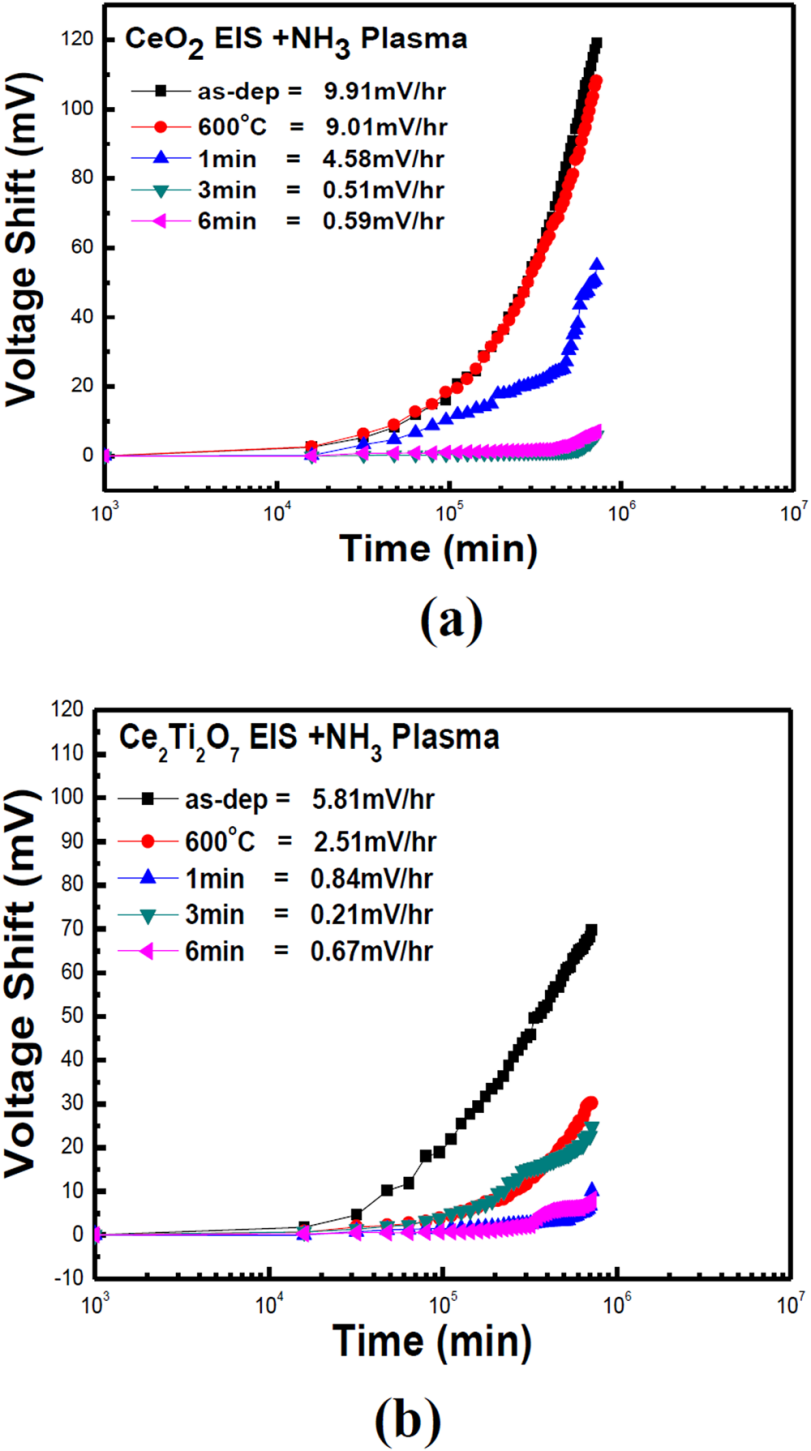
The drift voltage of (**a**) CeO_2_ and (**b**) Ce_2_Ti_2_O_7_ sensing membrane with NH_3_ plasma treatment in various conditions and then dipped in pH7 buffer solution for 12 hours.

Finally, the CeO_2_ membrane and the Ce_2_TiO_7_ membrane prepared in different plasma treatment conditions were tested for gate drift voltage, as shown in Fig. 9(a and b). Each of the samples were dipped in a pH7 buffer solution. In line with all the previous analyses, results reveal that the drift voltage shift could be reduced either by Ti doping or NH_3_ plasma treatment for 3 min. Moreover, the Ce_2_Ti_2_O_7_ membrane with NH_3_ plasma treatment had the least drift voltage of 0.21 mV/hr, signifying that Ti doping combined with NH_3_ plasma treatment could effectively enhance sensing capability. Moreover, to compare EIS membranes composed of various materials and treated with different treatment, the EIS pH biosensing devices incorporating ZnO, Gd_2_O_3_, Gd_2_TiO_7_ and Nb_2_O_5_ membranes are compared with CeO_2_ and Ce_2_TiO_7_ membranes as shown in Table 1 ^9, 32–34^.

**Table 1:** Comparison with pH sensing devices composed of various membranes with different treatments.

Sensing membrane	pH sensitivity (mV/pH)	Hysteresis voltage (mV)	Drift rate (mV/h)
ZnO with RTA	42.54	7.37	1.78
Nb_2_O_5_ with CF_4_	52.15	5.22	3.34
Gd_2_O_3_ with RTA	48.29	5.8	2.2
Gd_2_TiO_7_ with RTA	55.27	3.6	1.37
CeO_2_	34.37	27.7	9.91
CeO_2_ with NH_3_	48.62	7.8	0.51
Ce_2_Ti_2_O_7_	37.79	20.8	5.81
Ce_2_Ti_2_O_7_ with NH_3_	52.43	5.6	0.21

(RTA: rapid thermal annealing. CF_4_: CF_4_ plasma treatment. NH_3_: NH_3_ plasma treatment).

## Conclusions

In this study, CeO_2_ EIS biosensors incorporated Ti doping and NH_3_ plasma treatment were fabricated. Multiple material analyses suggest that the addition of Ti atoms to form Ce_2_Ti_2_O_7_ film could enhance grain growth and suppress dangling bonds. Furthermore, inclusion of N atoms by NH_3_ plasma treatment could reinforce crystallization and remove defects. Therefore, the sensitivity and linearity of the CeO_2_ EIS biosensor might be boosted and the hysteresis and the drift voltage could be reduced. CeO_2_-based EIS membranes with Ti doping and NH_3_ plasma treatment show promise for future industrial biosensing applications.

## Methods

Electrolyte-insulator-semiconductor (EIS) structures incorporating CeO_2_ and Ce_2_Ti_2_O_7_ sensing membranes were fabricated on 4 inch n-type (100) silicon wafers with a resistivity of 5–10 Ω-cm. After standard RCA cleaning, the samples were dipped into 1% hydrofluoric acid to etch native oxide from the surface. For the first type of samples, a 50 nm CeO_2_ film was deposited on the Si substrate by reactive radio frequency (rf) sputtering from a cerium target in diluted O_2_ ambient (Ar/O_2_ = 25 sccm/0 sccm). For the second group of samples, a 50 nm Ce_2_Ti_2_O_7_ sensing film was deposited by reactive radio frequency (rf) co-sputtering on an n-type silicon wafer, sputtered from a cerium target and a titanium target in diluted O_2_ ambient (Ar/O_2_ = 20 sccm/5 sccm). The rf power and chamber pressure were 100 W and 20 mTorr, respectively. After deposition, CeO_2_ and Ce_2_Ti_2_O_7_ were subjected to a post-NH_3_ plasma treatment in a plasma-enhanced chemical vapor deposition (PECVD) system with rf power of 30 W and a processing pressure of 500 mTorr for 1 min, 3 min and 6 min. Next, two types of samples were subsequently treated with rapid thermal annealing (RTA) using a conventional thermal annealing system under ambient N_2_ condition for 30 sec at a temperature of 600 °C. After that, the back-side contact of the Si wafer was deposited by Al film with a thickness of 300 nm. The sensing membrane size was defined through photolithographic processing under a photosensitive epoxy (SU8-2005, Micro-Chem). EIS structures were then fabricated on the copper lines of a printed circuit board (PCB) by using a silver gel to form conductive lines. Epoxy was utilized to separate the EIS structure and the copper line. The detailed EIS structure is illustrated in Fig. 1.
